# Chemical Stability and Bond Strength of Endodontic Sealers Exposed to Acidic, Alkaline and Protein‐Rich Environments: An In Vitro Investigation

**DOI:** 10.1111/aej.70038

**Published:** 2025-11-22

**Authors:** Walter Raucci‐Neto, Ronaldo Artacho Venter, Antônio Secco Martorano, Victória Gabriela Louzada, Carlos Eduardo Saraiva Miranda, Elias Daniel Covas Rodrigues, Larissa Moreira Spinola de Castro‐Raucci

**Affiliations:** ^1^ Department of Restorative Dentistry, School of Dentistry of Ribeirão Preto University of São Paulo Ribeirão Preto Brazil; ^2^ University of Ribeirão Preto—UNAERP Ribeirão Preto Brazil

**Keywords:** blood proteins, bond resistance, pH, sealers

## Abstract

The sealing performance of endodontic sealers may be influenced by environmental factors such as pH and blood‐derived proteins. This in vitro study evaluated the effects of pH variation and protein contamination on calcium ion (Ca^2+^) release and push‐out bond strength (BS) of a calcium silicate‐based sealer (Bio‐C Sealer) and an epoxy resin‐based sealer (AH Plus). Standardised bovine root dentine discs were immersed for 28 days in phosphate‐buffered saline (PBS) at pH 5, 7 or 12, with or without 10% fetal bovine serum (FBS). Bio‐C Sealer showed greater Ca^2+^ release and conductivity than AH Plus, with enhanced ion release in acidic and reduced in alkaline pH. Both sealers exhibited decreased BS in non‐neutral pH, though Bio‐C Sealer was more affected by FBS. These findings suggest that pH and protein exposure significantly alter sealer performance, with bioceramic materials being more sensitive to environmental changes than epoxy resin‐based sealers.

## 1 Introduction

Bioceramic sealers have gained attention for their biological and antimicrobial properties [1]. However, formulation adjustments were necessary to enhance flow and sealing, as traditional calcium silicate cements are dense and primarily used for repairs [2]. As a result, bioceramic sealers for obturation often show higher solubility and may present extended setting times [3]. Prolonged setting time of root canal sealers, influenced by environmental conditions, can compromise treatment by increasing solubility and reducing sealing capacity. Greater solubility may allow periradicular fluid penetration, raising local pH and destabilising the material, favouring microbial infiltration [4]. Therefore, to support clinical use with robust evidence, sealers are commonly evaluated based on physicochemical properties such as setting time, solubility, flow and radiopacity, which are critical to ensuring effective and durable endodontic sealing.

The solubility of root canal sealers is commonly evaluated in vitro using standardised protocols established by the International Organisation for Standardisation (ISO 6876:2012) and the American National Standards Institute/American Dental Association (ANSI/ADA Specification No. 57). These protocols measure solubility based on the specimen's mass loss after 24 h of immersion in distilled water. However, such conditions do not accurately reflect the clinical environment, which is often influenced by bleeding, inflammation, bacterial metabolites or prior intracanal medication with calcium hydroxide—all of which can alter local pH levels [5, 6]. Since sealers are placed in direct contact with periradicular tissues, the impact of pH and physiological fluids must be considered when assessing their physicochemical properties [3].

Studies indicate that bioceramic sealers exhibit significantly lower solubility in phosphate‐containing solutions that simulate biological fluids compared to distilled water [7], likely due to mineral precipitate formation on the material surface [8]. Furthermore, both calcium silicate‐based and epoxy resin‐based sealers may experience alterations in solubility and volumetric stability under acidic or alkaline pH conditions [3, 9]. These observations reinforce the importance of conducting complementary evaluations under clinically relevant conditions to improve the prediction of sealer performance in vivo.

Beyond conventional solubility testing, ionic release plays a central role in the clinical performance of calcium silicate‐based materials. Conductivity reflects the concentration and mobility of ions released from the sealer—particularly calcium and hydroxyl ions—which initiate mineral nucleation and drive the formation of apatite‐like phases at the material–fluid interface [10, 11]. Monitoring the electrical conductivity of immersion media therefore provides an indirect yet reliable approach to assessing a sealer's ion‐releasing behaviour, which is directly linked to its bioactivity and ability to promote biomineralization. This parameter complements pH and calcium‐release analyses, offering a more comprehensive understanding of the physicochemical mechanisms underpinning the biological performance of bioceramic sealers.

Because solubility and volumetric changes can directly affect sealing performance, it is also crucial to evaluate the bond strength of root canal sealers to dentine under conditions that closely simulate clinical application [3]. Over the long term, adequate bond strength is essential for maintaining a hermetic seal, preventing reinfection and supporting the continued healing of periapical tissues [12]. Acidic pH can reduce the bond strength of bioceramic sealers by compromising surface microhardness [13, 14]. However, previous studies focused solely on calcium silicate‐based sealers and used short storage periods, typically up to 7 days. Considering that bioceramic materials may require up to 30 days to fully set [15], and that some biofilms maintain acidic conditions over time, it is important to assess pH influence during both early and final stages of sealer setting to reflect clinical reality more accurately.

Few studies have investigated the effects of pH in physiological solutions on endodontic sealers, and those available have evaluated calcium silicate‐ and epoxy resin‐based materials [3, 9] without considering the potential influence of blood‐derived proteins. Proteins such as albumin and globulin may adsorb to the surface of calcium silicate sealers, forming a protective layer that reduces direct exposure to fluids and potentially lowers solubility [16]. Additionally, these proteins may chelate released ions, forming less soluble complexes and influencing the material's stability in biological environments [16].

Given that the bond strength of calcium silicate‐based sealers depends on sustained ionic exchange with dentine [10, 12], it is essential to assess whether blood proteins affect their interaction with root canal walls. This study tested the null hypothesis that different storage media would not significantly influence ionic release or bond strength of the evaluated sealers.

## 2 Materials and Methods

This laboratory study was approved by the Ethics Committee on the Use of Animals and was prepared in accordance with the Preferred Reporting Items for Laboratory Studies in Endodontology (PRILE) 2021 guidelines. The data that support the findings of this study are available on request from the corresponding author. The data are not publicly available due to privacy or ethical restrictions.

### 2.1 Experimental Design

The experimental factors for the ionic release analysis included: type of endodontic sealer at two levels (Bio‐C Sealer—Bio; AH Plus—AH), storage medium at six levels (PBS pH 5—PBS 5; PBS pH 7—PBS 7; PBS pH 12—PBS 12; PBS + serum pH 5—SERUM 5; PBS + serum pH 7—SERUM 7; PBS + serum pH 12—SERUM 12), and evaluation time at four levels (7, 14, 21 and 28 days). For the bond strength analysis, the factors were: type of sealer (Bio and AH) and storage medium (PBS 5, PBS 7, PBS 12, SERUM 5, SERUM 7 and SERUM 12).

A total of 120 samples were used, divided into two main groups according to the sealer (Bio or AH) and further subdivided based on the storage solution. Each experimental subgroup consisted of 10 replicates (*n* = 10), for both the ionic release and bond strength assessments.

Sample size calculation was performed using G*Power software, version 3.1. It was determined that a minimum of nine samples per group would be required to achieve a statistical power of 95.5%, assuming an alpha error of 0.05 and an effect size of 0.65.

### 2.2 Sample Preparation and Allocation According to Endodontic Sealer and Storage Medium

Sound bovine central incisors were selected based on root morphology, including shape, length, apical foramen closure, apical third diameter and canal volume. Roots were affixed to an acrylic plate using heated silicone adhesive (HK‐HM 60, Hikari, Brazil) and mounted on a precision cutting machine. Two dentine discs, each 2 mm thick, were sectioned per root. Canal diameters were measured buccolingually and mesiodistally with a digital calliper. Discs with diameters outside the 1.8–2.1 mm range were excluded. A total of 120 root dentine discs met the inclusion criteria and were selected for analysis.

Root dentine discs were stabilised on an acrylic resin plate (VIPI, Brazil) and mounted on a fixture (BioArt, Brazil) for standardised canal preparation. A water‐cooled air‐turbine handpiece was used, ensuring uniform preparations with a diameter of 2.3 mm and a depth of 2 mm. Discs were stored in deionised water before and after preparation.

After preparation, discs were affixed to glass slides with heated silicone adhesive and cleaned through irrigation with 2 mL of 2.5% NaOCl (Asfer, Brazil), delivered via syringe and aspirated with a Capillary Tip cannula (Ultradent, USA) connected to high‐power suction. Two subsequent rinses with 17% EDTA (Amazon, Brazil) were performed using the same method, followed by saline irrigation. Discs were randomly divided into two groups (*n* = 60). Group BIO was filled with Bio‐C Sealer (Angelus, Brazil) using the manufacturer's syringe and applicator. Group AH received AH Plus Jet (Dentsply Sirona, Germany), applied with the auto‐mix syringe system. Material details are presented in Table 1.

**TABLE 1:** Chemical composition and manufacturer details of the sealers.

Sealer	Manufacturer	Composition	Batch
AH Plus Jet	Denstisply Sirona	Paste A: Bisphenol A epoxy resin, Bisphenol F epoxy resin, calcium tungstate, zirconium oxide, silica, iron oxide pigments Paste B: Dibenzylamine, aminoadamantine, tricyclodecane‐diamine, calcium tungstate, zirconium oxide, silica, silicone	2011000844
Bio C Sealer	Angelus	Calcium silicate, calcium aluminate, calcium oxide, zirconium oxide, iron oxide, silicone dioxide and dispersing agent	62959

After filling, excess sealer was removed with a No. 24 spatula (Duflex, Brazil). After the initial setting time, each group was divided into six subgroups (*n* = 10) and immersed in 3.5 mL of experimental solutions.

Experimental solutions were prepared at 25°C, with pH adjusted every 72 h using 0.5 M NaOH or 0.1 M HCl and monitored with a calibrated pH meter. Phosphate‐buffered saline (PBS; Gibco, Thermo Fisher, USA) was prepared by dissolving 9.6 g of powder in 1 L of deionised water under agitation at 25°C. The pH was then adjusted to the target values of 5, 7 or 12.

The solution was filtered through a hydrophilic polyethersulfone (PES) membrane (30 mm, 0.22 μm; Jet Biofil, China). For FBS groups, 100 mL of FBS (Gibco, Thermo Fisher) was added to PBS to reach a 20% concentration. Solutions were stored at 4°C. Dentine specimens were immersed in 3.5 mL of each solution for 28 days at 37°C, with media renewed every 7 days.

### 2.3 Ionic Release Analysis

At each solution renewal, the immersion media were analysed for electrical conductivity, calcium ion (Ca^2+^) concentration and pH across the different storage periods, using a pre‐calibrated multiparameter device (AK151, AKSO Produtos Eletrônicos, São Leopoldo, RS, Brazil).

### 2.4 Bond Strength Test

Push‐out bond strength was tested using a universal machine (Instron EMIC 23‐5S, Instron, USA) at 1 mm/min. A 4 mm‐long plunger rod (1.8–2.0 mm diameter) was used, ensuring contact with at least 70% of the endodontic material surface without contacting the surrounding dentine.

The dislodgement force (*F*) required to displace the material was recorded in kilonewtons (kN) and converted into bond strength (*σ*) in megapascals (MPa) using the following formula: *σ* = *F*/*A*, where *A*, the material adhesion area, was obtained using the following formula: *A* = 2 *π* Re *h*, where Re = (Rm + rm)/2 and *A* = *π* (Rm + rm) *h*.

Interface and failure mode analysis was performed under a stereomicroscope (ZEISS Stemi 2000‐C, Germany) at 25× magnification. Failures were classified as adhesive (sealer–dentine interface), cohesive (within the material) or mixed (combination of adhesive and cohesive components) based on post–push‐out test images.

### 2.5 Statistical Analysis

Data normality and variance homogeneity were assessed using Shapiro–Wilk (*p* < 0.05) and Levene's tests. Two‐way ANOVA with Tukey's post hoc test (*α* = 0.05) was used to compare sealer type and solution. For ionic release, conductivity and calcium release, three‐way ANOVA was applied. Analyses were performed using SigmaPlot 12.0 (Systat Software, USA).

## 3 Results

### 3.1 Conductivity, Calcium Ion Release and pH

Bio‐C Sealer exhibited significantly higher values of conductivity, calcium ion release and pH than AH Plus, regardless of the storage medium or evaluation period (*p* < 0.05; Tables 2, 3, 4). Among the tested solutions, PBS at pH 5 (PBS 5) resulted in the highest conductivity and calcium release, while PBS at pH 12 (PBS 12) yielded the lowest values (*p* < 0.05). The presence of blood‐derived proteins significantly reduced both conductivity and calcium ion release compared to corresponding protein‐free solutions at the same pH (*p* < 0.05).

**TABLE 2:** Mean values and standard deviations of electrical conductivity (mS) of the solutions, according to the type of sealer and evaluation period.

	7 days		14 days	
	AH Plus	Bio C Sealer	AH Plus	Bio C Sealer
PBS 5	19.49 ± 0.46^Aa^	60.17 ± 0.07^Da^	16.55 ± 0.15^Ab^	45.82 ± 1.41^Cb^
PBS 7	15.77 ± 0.31^Ca^	50.73 ± 0.93^Ea^	16.75 ± 0.93^Aa^	36.85 ± 0.91^Db^
PBS 12	18.00 ± 0.41^Aa^	17.83 ± 0.82^1a^	17.40 ± 0.19^Aa^	20.68 ± 0.54^Fb^
SERUM 5	20.31 ± 0.50^Aa^	45.17 ± 0.07^Ha^	17.95 ± 0.43^Ab^	29.52 ± 0.73^Eb^
SERUM 7	17.07 ± 0.95^Ba^	47.53 ± 0.97^Ga^	21.70 ± 0.81^Bb^	26.22 ± 0.44^Eb^
SERUM 12	17.76 ± 0.49^Ba^	49.13 ± 0.94^Fa^	17.70 ± 0.38^Aa^	19.89 ± 0.79^Fb^

	21 days		28 days	
	AH Plus	Bio C Sealer	AH Plus	Bio C Sealer
PBS 5	15.99 ± 0.22^Ac^	24.07 ± 1.31^Cc^	16.62 ± 0.22^Ad^	17.09 ± 0.58^Bd^
PBS 7	16.13 ± 0.12^Aa^	20.38 ± 0.82^Dc^	16.67 ± 0.22^Aa^	17.14 ± 0.47^Bd^
PBS 12	18.15 ± 0.37^Ba^	20.00 ± 0.20^Db^	17.86 ± 0.39^Aa^	17.87 ± 0.54^Bc^
SERUM 5	17.00 ± 0.65^Ac^	18.29 ± 0.22^Dc^	16.98 ± 0.20^Ad^	16.82 ± 0.46^Bd^
SERUM 7	17.29 ± 0.73^Ac^	19.92 ± 0.72^Dc^	15.66 ± 0.44^Ac^	16.19 ± 0.57^Bd^
SERUM 12	18.00 ± 0.91^Ba^	17.14 ± 0.87^Ec^	17.14 ± 0.29^Aa^	17.42 ± 0.30^Bd^

*Note:* Identical uppercase letters indicate statistical similarity among cements within the same experimental period. Identical lowercase letters indicate statistical similarity among cements and time points within the same immersion solution.

**TABLE 3:** Mean values and standard deviations of calcium ion release (μg/mL) from the solutions, according to the type of sealer and storage period.

	7 days		14 days		21 days		28 days	
	AH Plus	Bio C Sealer	AH Plus	Bio C Sealer	AH Plus	Bio C Sealer	AH Plus	Bio C Sealer
PBS 5	2.20 ± 0.2^Aa^	177.81 ± 2.4^Ba^	2.04 ± 0.1^Aa^	162.69 ± 6.4^Bb^	1.57 ± 0.0^Aa^	135.63 ± 1.1^Bc^	1.32 ± 0.06^Aa^	87.63 ± 2.7^Bd^
PBS 7	1.71 ± 0.0^Aa^	158.43 ± 2.8^Ca^	1.68 ± 0.0^Aa^	140.43 ± 7.4^Cb^	1.42 ± 0.0^Aa^	116.67 ± 0.7^Cc^	1.32 ± 0.0^Aa^	70.43 ± 0.1^Cd^
PBS 12	1.97 ± 0.0^Aa^	133.35 ± 3.0^Da^	1.95 ± 0.0^Aa^	113.54 ± 1.2^Db^	1.64 ± 0.0^Aa^	85.39 ± 0.36^Dc^	1.33 ± 0.0^Aa^	50.39 ± 0.2^Dd^
SERUM 5	1.97 ± 0.0^Aa^	125.54 ± 2.3^Ea^	1.98 ± 0.0^Aa^	105.43 ± 0.6^Eb^	1.65 ± 0.0^Aa^	81.72 ± 0.52^Dc^	1.31 ± 0.0^Aa^	69.84 ± 0.9^Cd^
SERUM 7	1.66 ± 0.1^Aa^	117.86 ± 1.8^Fa^	1.65 ± 0.0^Aa^	84.59 ± 0.64^Fb^	1.38 ± 0.0^Aa^	70.35 ± 0.40^Ec^	1.29 ± 0.0^Aa^	48.87 ± 0.8^Dd^
SERUM 12	1.95 ± 0.0^Aa^	113.54 ± 1.2^Fa^	1.93 ± 0.0^Aa^	61.00 ± 0.43^Gb^	1.52 ± 0.0^Aa^	50.59 ± 0.29^Fc^	1.31 ± 0.0^Aa^	30.56 ± 0.2^Ed^

*Note:* Identical uppercase letters indicate statistical similarity among cements within the same experimental period. Identical lowercase letters indicate statistical similarity among cements and time points within the same immersion solution.

**TABLE 4:** Mean values and standard deviations of the hydrogen ion concentration (pH) of the solutions, according to the type of sealer and storage period.

	7 days		14 days	
	AH Plus	Bio C Sealer	AH Plus	Bio C Sealer
PBS 5	5.92 ± 0.11^Aa^	6.63 ± 0.01^Aa^	5.54 ± 0.02^Aa^	6.16 ± 0.01^Aa^
PBS 7	7.14 ± 0.02^Bb^	8.80 ± 0.01^Bb^	7.40 ± 0.20^Bb^	8.16 ± 0.26^Bb^
PBS 12	11.29 ± 0.52^Cc^	11.41 ± 0.44^Cc^	11.30 ± 0.49^Cc^	10.85 ± 0.36^Cc^
SERUM 5	5.76 ± 0.02^Aa^	5.83 ± 0.01^Aa^	5.46 ± 0.48^Aa^	5.69 ± 0.24^Aa^
SERUM 7	6.76 ± 0.02^Bd^	7.78 ± 0.01^Bb^	6.74 ± 0.02^Bb^	6.77 ± 0.01^Bb^
SERUM 12	8.33 ± 0.02^Ce^	10.80 ± 0.01^Cc^	10.44 ± 0.17^cf^	10.62 ± 1.00^Cc^

	21 days		28 days	
	AH Plus	Bio C Sealer	AH Plus	Bio C Sealer
PBS 5	5.77 ± 0.10^Aa^	6.00 ± 0.03^Aa^	5.71 ± 0.04^Aa^	5.81 ± 0.04Aa
PBS 7	7.06 ± 0.02^Bb^	7.65 ± 0.04^Bb^	7.32 ± 0.49^Bb^	7.09 ± 0.19Bb
PBS 12	11.12 ± 0.43^Cc^	10.69 ± 0.44^Cc^	11.12 ± 0.73^Cc^	11.01 ± 0.54^Cc^
SERUM 5	4.51 ± 0.33^AA^	5.44 ± 0.23^Aa^	5.35 ± 0.39^Aa^	5.47 ± 0.39Aa
SERUM 7	7.09 ± 0.11^Bb^	7.01 ± 0.28Bb	6.99 ± 0.25^Bb^	7.18 ± 0.39Bb
SERUM 12	11.00 ± 0.31^Cc^	8.37 ± 0.79^Cc^	11.08 ± 0.64^Cc^	11.20 ± 0.038^Cc^

*Note:* Identical uppercase letters indicate statistical similarity among cements within the same experimental period. Identical lowercase letters indicate statistical similarity among cements and time points within the same immersion solution.

All groups showed significant differences in pH values after contact with the sealers (*p* < 0.05). The highest pH levels were observed in PBS 12 and SERUM 12, and the lowest in PBS 5 and SERUM 5. Over time, conductivity, calcium ion release and pH progressively decreased in all groups, with the highest values at the initial time point and the lowest at the final evaluation (*p* < 0.05). However, pH remained stable after the first interval (*p* > 0.05).

In the AH Plus group, conductivity significantly decreased after 21 days, stabilising between days 21 and 28 (*p* < 0.05 and *p* > 0.05, respectively). Acidic and alkaline pH conditions promoted higher conductivity than neutral conditions (*p* < 0.05). For Bio‐C Sealer, conductivity decreased significantly as early as day 7 in samples stored at pH 5 and 7 (*p* < 0.05), while a reduction at pH 12 was also noted at day 7 (*p* < 0.05). In serum‐supplemented groups, conductivity declined significantly between days 7 and 14, with no further changes (*p* > 0.05).

Calcium ion release from AH Plus significantly decreased from the third evaluation period in all media (*p* < 0.05), remaining stable thereafter (*p* > 0.05). Higher release occurred at pH 5 and 12 compared to pH 7 (*p* < 0.05). Bio‐C Sealer showed progressive decreases from the first interval under all conditions (*p* < 0.05). Serum‐containing solutions led to lower calcium release than protein‐free media (*p* < 0.05), with no significant differences among protein‐free groups over time (*p* > 0.05).

pH values remained stable in all groups, except AH Plus in SERUM 12, which showed a significant increase after the initial time point (*p* < 0.05; Table 4).

### 3.2 Bond Strength and Failure Pattern

Tukey's post hoc test showed that AH Plus presented significantly higher bond strength (BS) than Bio‐C Sealer in all storage solutions (*p* < 0.01; Table 5). For both sealers, samples stored in PBS at pH 7 exhibited the highest BS values (*p* < 0.05). Exposure to blood proteins in acidic or alkaline environments resulted in significantly lower BS compared to protein‐free solutions of the same pH (*p* < 0.05), whereas storage in neutral pH with or without proteins showed no significant difference (*p* > 0.05).

**TABLE 5:** Mean values and standard deviations of bond strength (MPa) of the sealers under different storage media.

	AH Plus	Bio C Sealer
PBS 5	4.12 ± 0.20^Bc^	2.93 ± 0.24^Dc^
PBS 7	5.44 ± 0.31^Ab^	3.97 ± 0.27^Cc^
PBS 12	3.89 ± 0.35^Cb^	2.52 ± 0.24^Dc^
SERUM 5	4.06 ± 0.41^Ba^	1.63 ± 0.45^Ea^
SERUM 7	5.26 ± 0.48^Aa^	2.37 ± 0.43^Da^
SERUM 12	3.91 ± 0.43^Ca^	1.80 ± 0.43^Ea^

*Note:* Identical uppercase letters indicate statistical similarity for comparisons within the same column. Lowercase letters denote statistically significant differences across comparisons made within the same row.

In the AH Plus group, BS values in acidic and alkaline solutions did not differ significantly (*p* > 0.05), regardless of fetal bovine serum presence, but both conditions resulted in lower BS than neutral pH (*p* < 0.05). For Bio‐C Sealer, samples stored in alkaline solutions had significantly lower BS than those stored at neutral or acidic pH (*p* < 0.05), with no differences between the latter two (*p* > 0.05). Fetal bovine serum significantly reduced BS only in the Bio‐C group (*p* < 0.05).

**FIGURE 1:**
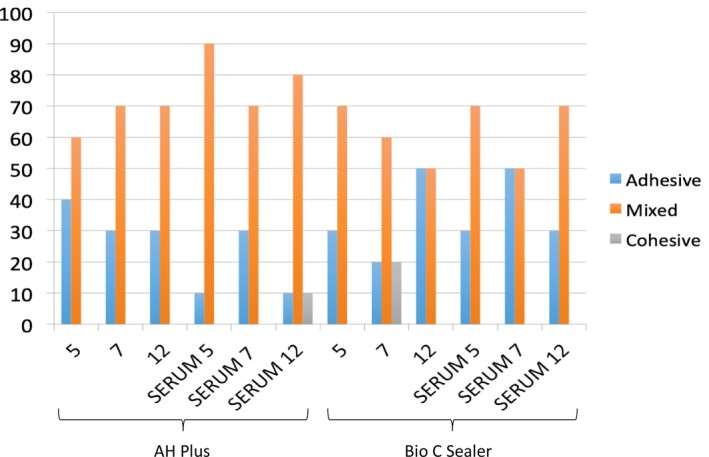
Failure patterns of the endodontic sealers under different storage media.

Failure pattern analysis showed a predominance of mixed failures across most groups, except for Bio‐C Sealer stored in PBS 12 and SERUM 7, which showed a balanced distribution between mixed and adhesive failures (Figure 1).

## 4 Discussion

Successful endodontic treatment relies on techniques and materials that prevent fluid infiltration and microbial growth. Therefore, assessing the physicochemical and mechanical performance of sealers in clinically relevant environments is essential. The results of this study led to the rejection of the null hypothesis, as significant differences in ionic release and bond strength were observed between calcium silicate‐based and epoxy resin‐based sealers, as well as among the various storage media tested.

The setting environment directly influences the physicochemical behaviour of sealers. Solubility and sealing capacity are critical to endodontic success [3], and pH can significantly affect surface properties and performance [17]. Most in vitro studies use distilled water, which poorly replicates clinical conditions [9]. To overcome this, the present study simulated realistic scenarios by immersing samples in solutions with different pH levels, with or without blood protein supplementation.

Endodontic sealers are designed to create a hermetic seal and prevent fluid ingress into the root canal system, including through anatomical complexities such as apical foramina and dentineal wall irregularities. Achieving an effective seal is essential to limit the extrusion of residual bacteria into the periapical tissues and promote periapical healing [18, 19].

A key feature of bioceramic sealers is their ability to release ions, which promotes the formation of crystalline precipitates upon contact with body fluids [10]. In clinical scenarios where the apical foramen is patent or sealer extrusion occurs, these mineral structures may interact with periapical tissues and contribute to stimulating mineralised matrix deposition by osteogenic and odontogenic cells [20, 21]. Within the root canal system, however, their biological significance is mainly associated with apatite nucleation at the sealer–dentine interface. This process enhances chemical bonding, improves interfacial adaptation and reduces the risk of microleakage [22]. Consequently, biomineralization strengthens the sealer's integration with root dentine, providing long‐term stability to the filling material and indirectly supporting periapical healing.

For the present study, AH Plus and Bio‐C Sealer were selected due to their distinct chemical compositions and physicochemical behaviours. These differences provided a relevant basis for investigating their performance under experimental conditions designed to simulate the clinical environment.

Conductivity and calcium ion release results revealed distinct behaviours between the evaluated sealers. Although both materials showed a reduction in these parameters over the 28‐day period, AH Plus consistently exhibited significantly lower values than the bioceramic sealer. This difference is likely due to the lower solubility of epoxy resin‐based sealers, as supported by previous studies [2, 23]. In contrast, the higher solubility of the bioceramic material may be associated with its extended setting time, which can reach 30 days [15]. Calcium ion release, resulting from calcium hydroxide dissociation during hydration and setting [9], may promote hydroxyapatite formation but also lead to volumetric instability [3].

Regarding the influence of immersion solutions, Bio‐C Sealer samples stored in alkaline pH exhibited reduced calcium ion release and electrical conductivity compared to those in neutral pH. Under neutral conditions, bioceramic sealers release calcium and silicon ions, enabling hydroxyapatite formation through interaction with phosphate ions in solution [12]. This promotes controlled ion release and mineralization at the sealer–dentine interface [12, 17]. In contrast, exposure to alkaline environments—such as those resulting from chronic inflammation or alkaline irrigants—may alter ion dissociation kinetics [24]. The high affinity of hydroxyl ions (OH^−^) for calcium can lead to the formation of stable ionic complexes, reducing the concentration of free calcium ions detected in solution [25] and potentially modifying the material's behaviour.

In the presence of fetal bovine serum (FBS), Bio‐C Sealer showed reduced conductivity and calcium ion release, likely due to protein adsorption impairing ionic exchange [25]. Although bioceramic sealers perform well in moist environments [26], direct blood contact may hinder ion release and interfere with chemical interactions at the sealer–dentine interface, affecting physicochemical performance [27].

The push‐out test was selected to evaluate the displacement resistance of the sealers because of its reproducibility and reliability [10, 28]. One limitation of this method, however, is the challenge of standardising specimen positioning, as misalignment may affect the accuracy of the measured bond strength. In the present study, specimen orientation was carefully standardised so that the canal walls were parallel to the direction of the applied load, and the plunger was precisely centred on the filling material without contacting the surrounding dentine [10], thereby increasing the reliability of the retention assessment.

This study exclusively used sealers without gutta‐percha to ensure full dentine coverage and improve standardisation. Although gutta‐percha is common in obturation, it does not bond to dentine and depends on the sealer for sealing [29]. This approach also simulated clinical scenarios, such as wide apical foramina or resorption, where only sealers are used [17, 29].

Optimal adhesion between the sealer and root dentine is essential to prevent fluid ingress and ensure long‐term endodontic success [28]. Stability of the adhesive interface is also critical during retreatment or post‐placement, where mechanical stress may dislodge the sealer [29]. This study's bond strength tests aimed to assess how the physicochemical setting environment influences sealer–dentine interaction, aiding in material selection for improved clinical outcomes.

In this study, both the chemical composition of the sealers and the setting environment significantly affected bond strength. AH Plus consistently exhibited higher bond strength than Bio‐C Sealer under all storage conditions. This finding is consistent with previous research and may be attributed to its low solubility, high dimensional stability and favourable flow, which help minimise marginal gaps and improve adhesion to dentine [19, 23]. For AH Plus, pH played a key role, with the highest bond strength observed under neutral conditions. Acidic and alkaline environments led to reduced values, likely due to increased solubility and associated volumetric changes, as noted by Ferreira et al. [9]. This interpretation is further supported by the lower conductivity values recorded in the neutral pH group.

Moreover, the performance of Bio‐C Sealer was particularly affected by the presence of fetal bovine serum, which significantly reduced its bond strength to root dentine. Previous studies have suggested that protein adsorption from blood components can interfere with hydroxyapatite mineralization and form a surface layer that hinders intimate contact between the sealer and dentine [16]. These findings are consistent with the reduced conductivity and calcium ion release values observed in the present study, as well as with previous reports [30, 31]. Considering the lower influence of blood proteins on AH Plus, these results suggest that, even under conditions of blood contamination, the epoxy resin‐based sealer may exhibit superior clinical performance.

Whereas the adhesion of bioceramic sealers primarily depends on biomineralization at the sealer–dentine interface, epoxy resin‐based sealers such as AH Plus adhere through covalent bonding with amino groups in the exposed collagen of intertubular dentine [32]. The present findings suggest that this chemical interaction is largely unaffected by the presence of blood proteins.

Additionally, exposure to alkaline pH significantly reduced the bond strength of Bio‐C Sealer compared to that observed under neutral or acidic conditions. These results corroborate the conductivity and calcium ion release data, suggesting that an alkaline setting environment may interfere with the hydration and setting reactions of the material. As shown in earlier studies [3, 9], such alterations in setting kinetics may increase solubility and compromise adhesion. Therefore, these findings suggest that the application of calcium hydroxide‐based intracanal medicaments may adversely influence the physicochemical or clinical performance of bioceramic sealers. Moreover, future investigations should consider exploring pH neutralisation protocols following the use of calcium hydroxide medicaments, aiming to optimise the conditions for obturation when employing such sealers.

A clear correlation was observed between bond strength and failure mode. Mixed failures were more prevalent in groups with higher bond strength, while adhesive failures predominated in groups with reduced adhesion—particularly among Bio‐C Sealer samples exposed to blood proteins.

Although this study aimed to simulate clinically relevant conditions, it was conducted in vitro under standardised protocols. In vivo, biological factors such as immune response and tissue healing may affect material performance. Solution renewal simulated persistent infection or calcium hydroxide leakage. A 30‐day period was chosen to ensure full setting of the bioceramic sealer and mature property assessment.

The findings of this study have relevant clinical implications. The physicochemical properties and bond strength of endodontic sealers are influenced by environmental conditions, especially pH and the presence of blood proteins. These results support evidence‐based sealer selection tailored to each clinical scenario. AH Plus showed superior bond strength and dimensional stability, reinforcing its status as the gold standard in cases requiring a reliable seal. However, its lower biocompatibility may limit use in specific contexts, highlighting the potential of bioceramic sealers like Bio‐C Sealer, which promote bioactivity and ion release for tissue repair [33]. Still, reduced performance in alkaline environments or with blood proteins underscores the importance of context‐driven material choice.

Further research should focus on the development of novel endodontic sealers that combine high mechanical performance with improved biological interactions, particularly under conditions of altered pH and biological fluid exposure. Studies exploring the long‐term clinical performance of these materials in vivo are also warranted. Advancements in this area will contribute to the design of next‐generation sealers with optimised sealing ability, biocompatibility and dimensional stability, ultimately enhancing the predictability and longevity of endodontic treatment outcomes.

## 5 Conclusion

The results of this study indicate that Bio‐C Sealer showed higher ionic release but lower bond strength than AH Plus across all storage media. Blood proteins negatively affected both properties in Bio‐C Sealer. Additionally, acidic and alkaline pH conditions reduced the bond strength of both endodontic sealers.

## Author Contributions

Walter Raucci‐Neto: conceptualisation, methodology, investigation, data analysis, interpretation, writing. Ronaldo Artacho Venter: teeth collection, conceptualisation, methodology, investigation, reviewing. Antônio Secco Martorano: conceptualisation, methodology, data analysis. Victória Gabriela Louzada: conceptualisation, methodology, investigation. Carlos Eduardo Saraiva Miranda: methodology, investigation, reviewing. Elias Daniel Covas Rodrigues: conceptualisation, methodology, reviewing. Larissa Moreira Spinola de Castro‐Raucci: conceptualisation, methodology, interpretation, reviewing. All authors are in agreement with the manuscript.

## Funding

This work was supported by the Coordination for the Improvement of Higher Education Personnel (CAPES—Finance Code 001), Brazil.

## Conflicts of Interest

The authors declare no conflicts of interest.

## Data Availability

The data that support the findings of this study are available on request from the corresponding author. The data are not publicly available due to privacy or ethical restrictions.
